# Myeloidcells in the immunosuppressive microenvironment in glioblastoma: The characteristics and therapeutic strategies

**DOI:** 10.3389/fimmu.2023.994698

**Published:** 2023-02-27

**Authors:** Boyuan Huang, Jin Zhang, Wenjing Zong, Sisi Chen, Zhitao Zong, Xiaojun Zeng, Hongbo Zhang

**Affiliations:** ^1^ Department of Neurosurgery, Capital Medical University Electric Power Teaching Hospital/State Grid Beijing Electric Power Hospital, Beijing, China; ^2^ Department of Neurosurgery, The First Affiliated Hospital, Jinan University, Guangzhou, China; ^3^ Institute of Chinese Materia Medica, China Academy of Chinese Medical Sciences, Beijing, China; ^4^ Department of neurosurgery, Jiujiang Hospital of Traditional Chinese Medicine, Jiujiang, China; ^5^ Department of Neurosurgery, Shenzhen Key Laboratory of Neurosurgery, Shenzhen Second People’s Hospital, The First Affiliated Hospital of Shenzhen University, Shenzhen, China; ^6^ Department of Neurosurgery, The Second Affiliated Hospital of Nanchang University, Nanchang, China

**Keywords:** myeloid cells, immunosuppressive, tumor microenvironment, glioblastoma, immunotherapies

## Abstract

Glioblastoma (GBM) is the most common and lethal malignant tumor of the central nervous system in adults. Conventional therapies, including surgery, radiotherapy, and chemotherapy, have limited success in ameliorating patient survival. The immunosuppressive tumor microenvironment, which is infiltrated by a variety of myeloid cells, has been considered a crucial obstacle to current treatment. Recently, immunotherapy, which has achieved great success in hematological malignancies and some solid cancers, has garnered extensive attention for the treatment of GBM. In this review, we will present evidence on the features and functions of different populations of myeloid cells, and on current clinical advances in immunotherapies for glioblastoma.

## 1 Introduction

Glioblastoma (GBM), accounting for more than 50% of diagnosed intracranial glioma and approximately 12% of all brain tumors, is the most common and lethal malignant tumor of the central nervous system (CNS) in adults (1–3). GBM is of a highly aggressive nature, with fast tumor growth, diffuse tumor invasiveness, and high levels of resistance to conventional therapies (4). It occurs frequently in people aged 50– 60 years and is more prevalent in men (1). GBM is traditionally classified into two groups: primary GBM arising *de novo*, of which 90% have a wild-type isocitrate dehydrogenase (IDH) profile, and secondary GBM, which develops from low-grade glioma (5). On the basis of a genomic profile, primary GBM can be further categorized into three subgroups: classical (CL) GBM, with abnormal expression of epidermal growth factor receptor (EGFR) and homozygous deletion of cyclin-dependent kinase inhibitor 2A (*CDKN2A*); proneural (PN) GBM, which is characterized by the amplification of platelet-derived growth factor receptor alpha (PDGFRα) and a tumor protein p53 (*TP53*) mutation; and mesenchymal (MES) GBM featuring co-mutated phosphatase and tensin homolog (*PTEN*) and *TP53* tumor suppressor genes and the lack of the neurofibromatosis type 1 (*NF1*) gene function (5–8). It has been demonstrated that the MES subgroup showed the shortest median survival of 11.5 months, compared with 17 months for the PN subgroup and 14.7 months for the CL subgroup (6). Secondary GBM harbors *IDH1* and *IDH2* mutations, which can convert α-ketoglutarate (α-KG) to the oncometabolite 2-hydroxyglutarate (2-HG) to initiate tumorigenesis (6).

The current standard treatment for GBM, comprising a combination of maximal surgical resection, radiation, and chemotherapy, has limited success in improving the prognosis of patients, who are given only 15 months of median survival and a 5-year survival rate of less than 5% (9–11). Thus, opportunities and challenges remain in finding more efficient treatments for GBM. Currently, immunotherapy, which has obtained great success in treating hematopoietic malignancies, malignant melanoma, and some solid tumors, has garnered extensive attention for treating GBM (12). Current immunotherapy mainly focuses on the investigation of vaccine therapy, adoptive T-cell therapy, immune checkpoint inhibitors (ICIs) therapy, and oncolytic virus therapy. Unfortunately, immunotherapeutic successes in GBM are still lacking (13). Multifarious factors have attenuated the immunotherapeutic efficiency for GBM. It has been long recognized that the brain is an immune-privileged organ based on the restriction from the brain–blood barrier (BBB) (14). However, this concept has been challenged by the existence of functional lymphatic vessels and varied types of leukocytes in the CNS. Today, the brain is considered immunologically “distinct” rather than “privileged” (15). Moreover, a consensus has been reached on the idea that the immunosuppressive tumor microenvironment (TME) of GBM plays a crucial role in the resistance to current treatment. The GBM microenvironment is intensively infiltrated with a variety of myeloid cells, including bone marrow-derived macrophages (BMDMs), microglia, myeloid-derived suppressor cells (MDSCs), and neutrophils. Recent flow cytometric and single-cell analyses have revealed that glioma cells (40.5%) and myeloid cells (45%) constitute the tumor mass, both of which can secrete cytokines and metabolites to suppress tumor-infiltrating lymphocyte (TIL) function (16, 17). Thus, the lack of TILs and abundant immunosuppressive myeloid cells constitute a significant barrier against immunotherapy efficacy in GBM. A recent study that combined single-cell analysis and spatial transcriptomics identified reactive-hypoxia and reactive-immune regions in the GBM TME. These regions were found to be enriched with memory and exhausted T cells as well as myeloid cells, suggesting a local enhanced immunosuppression (18). Moreover, through analysis of cell-to-cell interactions among different cancer and immune cells, researchers found that bidirectional signaling between glioma cells and myeloid cells, and myeloid cells and T cells is much more abundant than signaling between T cells and glioma cells, indicating that the myeloid cells are major conduits of cell-to-cell interaction in the GBM microenvironment (19). Accordingly, investigating the features and functions of these myeloid cells as well as their interplay with cytotoxic lymphocytes could provide insight into the establishment of the TME, which could be applied to the development of immunotherapies against GBM. In this review, we will present evidence on the features and functions of different populations of myeloid cells, including those of BMDMs, microglia, MDSCs, and neutrophils. Moreover, we will also present evidence of current advances in immunotherapies for GBM, including vaccine therapy, adoptive T-cell therapy, ICIs therapy, and oncolytic virus therapy.

## 2 Myeloid cells in GBM

### 2.1 Microglia and macrophages in GBM

Tumor-associated macrophages (TAMs), which originate from bone marrow-derived monocytes, represent one of the most prominent populations in the tumor stroma. In GBM, the TAMs, also called glioma-associated macrophages/microglia (GAM), are composed of BMDMs and microglia, which constitute the majority of tumor-invading myeloid cells and approximately 30% of the tumor mass (20–22). Whereas microglia, derived from yolk sac progenitors, mainly reside in peritumoral regions in GBM, BMDMs, derived from hematopoietic stem cells (HSCs), are preferentially located in perivascular regions in GBM (23–25). Traditionally, microglia and BMDMs share the same marker of CD11b; however, microglia are distinguished by low levels of expression of CD45. Considering the phenomenon that activates microglia can rapidly up-regulate CD45 expression, two new markers are proposed to distinguish microglia from macrophages: the transmembrane protein 119 (TMEM119) and purinergic receptor P2RY12 (26–28). Typically, microglia and macrophages can be stimulated into two different polarizations *in vitro*: the pro-inflammatory M1 phenotype and the anti-inflammatory M2 phenotype. In cancer, this M1/M2 classification has also been used for decades to evaluate whether microglia and macrophages have anti-tumor (M1) or tumor-promoting (M2) characteristics (29, 30). However, compelling evidence suggests that this categorization does not apply to *in vivo* conditions because it only describes the extremes of a broader spectrum of functional states (31). This is supported by microarray analysis of GAMs isolated from GL261-implanted C57BL/6 mouse brains, which showed a different profile from the M1 and M2 phenotypes, including a mixture of M1- and M2-specific genes (32). Moreover, genetic analysis of TAMs from GBM patients demonstrated that these cells exhibited diverse immunological functions as a non-polarized M0 phenotype (33). However, a recent study has argued that GAM subtypes *in vivo* are not directly equivalent to *in vitro*-defined M0-, M1-, or M2-like macrophages (34). Recently, a single-cell analysis of GBM multiregionally and multidimensionally identified four molecular subtypes of microglia: MC1 (i-Mic), with high levels of expression of activated microglia markers and TNF, IL1B, and NFKBIZ; MC2 (h-Mic), with the highest level of expression of the homeostatic microglia marker CST3; MC6 (AP-Mic), with expression of both microglia and macrophage markers; and MC7 (a-Mic), identified by differential levels of expression of SPRY1, PYRY13, and microglia activation markers (19). Moreover, a further investigation of the relationship between the gene signature and patient survival showed that MC2 and MC7 were related to better survival, indicating an anti-tumorigenic role in the TME. Accordingly, a comprehensive and functional classification system is still needed to describe the diverse states of GAMs in GBM.

In the TME, the GAMs predominantly exhibit an anti-inflammatory M2 polarization and reduced phagocytic activity (35, 36). GAMs can be driven to the TME by a variety of chemokines, including CXCL12, CCL2, CX3CL1, MCP-1, MCP-3, glial cell-derived neurotrophic factor (GDNF), and osteopontin released by neoplastic cells (36–41). Moreover, GBM cells can induce GAM invasion by expressing the macrophage-stimulating factor CSF-1, which regulates macrophage proliferation and differentiation, and guides the macrophages to polarize toward the protumorigenic M2 phenotype (42). Recently, the secretion of periostin from glioma stem cells (GSCs) has also been demonstrated to support macrophage recruitment and transition into an M2 phenotype, and the deletion of POSTN in GSCs resulted in a dramatic reduction of GAM density and GBM growth, as well as an increased survival rate of mice bearing GSC-derived xenografts (43). Once infiltrated into the TME and induced by GBM into a protumoral phenotype, GAMs can promote the tumor cells’ growth and invasion through multifarious pathways. The GBM-associated microglia can secrete epidermal growth factor (EGF) to facilitate GBM cell migration and invasion through binding to the epidermal growth factor receptor (EGFR) on GBM cells (42). Moreover, the GAMs can release the tumor growth factor beta (TGF-β) 1 to bind the TGF-β receptor (TGFBR) 2 (TGFBR2) on GBM cells, which can promote tumor invasion (44). Furthermore, GBM-associated microglia can up-regulate matrix metallopeptidase (MMP14), which can cleave into an inactive form of MMP2 and thus facilitate the invasion of glioma cells into the brain parenchyma by metalloproteinase-mediated degradation of the extracellular matrix (45). Although microglia are the most powerful phagocytes in the TME, their phagocytic function is impaired by GBM cells through rendering microglia as an anti-inflammatory, antiphagocytic M2 phenotype (46). For example, GBM cells can secrete molecules, such as CSF1 and CSF2, to induce the shift of GAMs toward a protumoral phenotype and thus create a favorable TME for GBM growth (42, 46). Moreover, GBM cells can also inhibit microglial phagocytosis by overexpressing sialic acid-rich glycoproteins and up-regulating CD47, which often acts as an antiphagocytic protein in cancer cells (47–49).

Taken together, GAMs are plastic and heterogeneous in their profiles and functions. The recruitment of GAMs is regulated by multiple factors. Induced into a protumoral phenotype in the TME, GAMs play a critical role in promoting the proliferation, invasion, and function of GBM cells.

### 2.2 Myeloid-derived suppressor cells in GBM

Myeloid-derived suppressor cells (MDSCs), presenting at very low frequencies of peripheral blood mononuclear cells, are a heterogeneous cluster of immature myeloid cells (IMCs) that can be differentiated into macrophages, dendritic cells (DCs), and granulocytes under physiological conditions (31, 50, 51). In pathological conditions such as GBM, the IMCs are driven to differentiate into MDSCs not only in the tumor bed but also in the peripheral blood (51). Human MDSCs are generally characterized by co-expressions of the myeloid differentiation markers, CD33 and CD11b, and with negative expression of markers of mature lymphoid and myeloid cells, such as human leukocyte antigen DR(HLA-DR) and major histocompatibility complex (MHC) class II molecule (52). In the human body, there are three major groups of MDSCs: early-stage MDSCs (eMDSCs), characterized by CD33^+^ and Lin^–^; granulocytic or polymorphonuclear MDSCs (PMN-MDSCs), defined as CD11b^+^Ly6G^+^Ly6C^low^ and CD33^+^CD15^+^CD14^–^HLA-DR^low/–^; and monocytic MDSCs (M-MDSCs), featuring CD11b^+^CD33^+^CD14^+^ Ly6C^high^ and Ly6G^–^D15^–^HLA-DR^low/–^. Although PMN-MDSCs are morphologically and phenotypically analogous to neutrophils, M-MDSCs are similar to monocytes (52–54).

Increasing evidence demonstrates that MDSCs are elevated in the peripheral blood of GBM patients (55). In GBM patients, elevated MDSC accumulation at the time of recurrence predicts poor prognosis, whereas the reduction of MDSCs is correlated with extended survival (55). With respect to the subgroups of MDSCs, although both PMN- and M-MDSCs were found to be increased in the peripheral blood of GBM patients, only PMN-MDSCs were found to be increased in the TME of GBM patients (56). Moreover, the two subsets of MDSCs were found to expand and thus drive immune suppression in a sex-specific manner (57). For example, the proliferation of M-MDSCs was predominant in tumors from male GBM patients, whereas PMN-MDSCs were enriched in female GBM patients’ blood, with significant relation to poor prognosis (57). In addition, M-MDSCs have also been claimed to constitute the major population of MDSCs within the glioma microenvironment. In accordance with these findings, the improvement in survival induced by the depletion of M- and PMN- MDSCs in GBM mouse models was also displayed in a sex-specific manner (57).

In GBM, MDSCs play critical roles in the sustenance of tumor growth, invasion, vascularization, and immunosuppression (58). Several mechanisms have been demonstrated to affect the accumulation of MDSCs in the GBM TME and the promotion of their immunostimulatory phenotype, which enables them to exert their immunosuppressive effects. GBM cells can recruit MDSCs from the bone marrow through up-regulation of indoleamine 2,3-dioxygenase (IDO1) and CD200, as well as secretion of macrophage migration inhibitory factor (MIF) and CCL20 (59–61). Moreover, they can also overexpress hypoxia-inducible factor 1 alpha (HIF1α) and vascular endothelial growth factor (VEGF) to promote the recruitment of MDSCs by converting extracellular ATP to 50-AMP (62). After accumulation in the TME, the proliferation and functions of MDSCs are controlled by various factors, such as CCR2, IL-6, IL-10, IL-8, IL-12, TGF-β, M-CSF, galectin-1,GM-CSF, and INF-γ, which can stimulate JAK, STAT1, STAT3, STAT6, CCAAT/enhancer-binding protein (C/EBPs), S100A8, S100A9, and prostaglandin E2 (PGE2) in MDSCs to promote their proliferation (63–65).

MDSCs can contribute to immunosuppression by suppressing the anti-tumor activity of cytotoxic T cells, inhibiting the functions of natural killer (NK) cells, macrophages, and DCs, and introducing the accumulation of regulatory T cells (Tregs) and immune-suppressive regulatory B cells (Bregs) (66–69). In glioma rat models, MDSCs can produce NO, iNOS, ARG1, IDO, ROS, and peroxynitrite (PNT), and secrete anti-inflammatory cytokines such as IL-10 and TGF-β to suppress T-cell proliferation, induce T-cell apoptosis, and attenuate T-cell cytotoxic function (70–73). In addition, MDSCs can overexpress ICIs such as the program death 1 ligand (PD-L1), which binds to its receptor PD1 to cause T-cell exhaustion (74). In accordance with T cells, MDSCs can also inhibit the cytotoxicity of NK cells. For example, the TGF-β1 on MDSCs can attenuate cytotoxicity of NK cells by down-regulating the expression of INFγ and activating the receptor NKGD. Moreover, a subset of MDSCs in mice were found capable of suppressing perforin production of NK cells by regulating the Janus Kinase-Signal transducers and activators of transcription (JAK-STAT) pathway (67, 75). With respect to DCs, MDSCs-derived NO can suppress the antigen presentation from DCs to CD4^+^ T cells, which can be abrogated by treatment of MDSC with iNOS inhibitors (76). In addition, MDSC can suppress TLR-ligand-induced IL-12 production of DCs *via* IL-10 secretion and inhibit the T-cell stimulatory function of DCs (77).

Apart from the inhibition of T cells, NK cells, and DCs, MDSCs can also exert an immunosuppressive function by recruitment and regulation of Tregs, Bregs, and M2 macrophages. MDSCs can release cytokines such as IL-10, INFβ, and TGF-β to drive naive T cells to differentiate into Tregs, and chemokines such as CCL3, CCL4, and CCL5 through CCR5 to recruit Tregs into the TME (78, 79). In return, Tregs can affect the survival and/or the proliferation of MDSCs and their direct interactions can suppress the functions and promote the apoptosis of CD8^+^ T cells (80). In addition, MDSCs can recruit Bregs, which account for approximately 40% of the immune-infiltrating cells in GBMs, into the TME to exert their immunosuppressive functions. Bregs can inhibit CD8^+^T-cell cytotoxic functions by overexpressing PD-L1 through the uptake of MDSC-derived microvesicles containing PD-L1. In addition, Bregs can also up-regulate CD155 expression and secrete immunosuppressive cytokines IL-10 and TGF-β to suppress CD8^+^ T-cell cytotoxicity (81). With respect to macrophages, the presence of MDSCs in tumors is correlated with a declined level of the anti-inflammatory M1 macrophages and an elevated level of pro-tumor M2 macrophages (77). In addition, MDSC-derived IL-10 can inhibit macrophages from secreting IL-6 and TNF-α (82, 83). Furthermore, MDSCs can down-regulate the MHC class II expression on macrophages to suppress their antigen presentation functions (82, 83). Lastly, it was demonstrated that MDSCs themselves can differentiate into TAMs when faced with hypoxic conditions in the TME (84).

**Figure 1:**
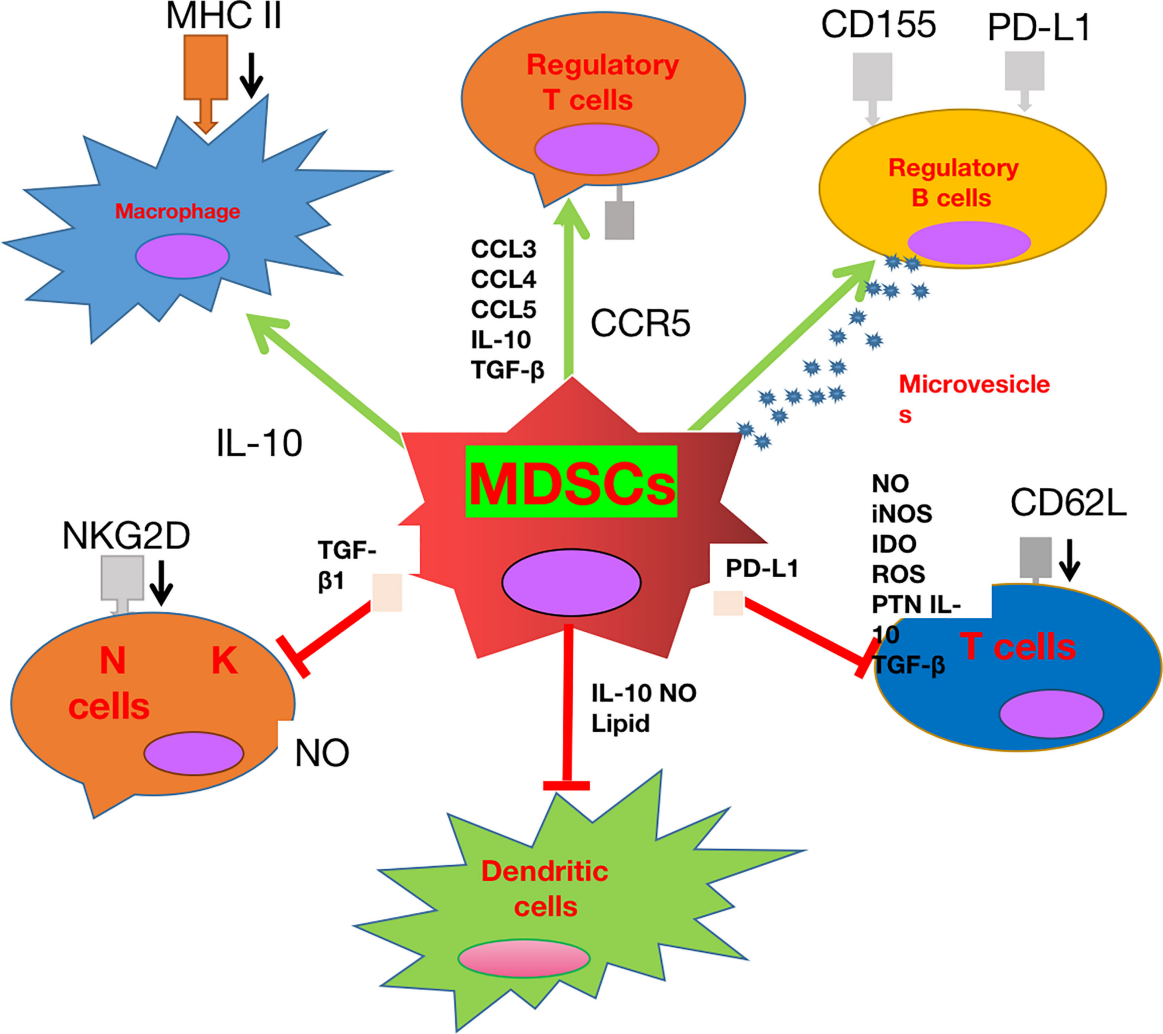
The role played by myeloid-derived suppressor cell (MDSC) glioblastoma (GBM). MDSCs can not only inhibit the cytotoxic effect and antigen presentation functions of T cells, natural killer (NK) cells, and dendritic cells (DCs), but also promote the recruitment and stimulation of immune-suppressive M2 macrophages, regulatory T cells (Tregs), and regulatory B cells (Bregs).

In summary, MDSCs play a crucial role in sustaining immunosuppression in the TME and their recruitment into the TME is regulated by GBM cells and other factors. MDSCs contribute to the immunosuppressive TME not only by suppressing the maturation, stimulation, and cytotoxic functions of T cells, DCs, and NK cells, but also by supporting the recruitment and inhibitory functions of Tregs, Bregs, and M2 macrophages. Further investigations will be carried out to explore the detailed mechanisms (**Figure 1**).

### 2.3 Neutrophils in GBM

Neutrophils, as the first respondent against pathogens, are the most abundant circulating leukocytes in humans, with multiple functions including phagocytosis, secretion of reactive oxygen species and granules, and formation of neutrophil extracellular traps (85). Tumor-associated neutrophils (TANs) display two typical phenotypes of polarization, which can switch into one another during tumor progression: the anti-tumoral N1 polarization elicited mainly by IFN-β and the protumoral N2 polarization induced by G-CSF, TGF-β1, and IL-6 (86). The number of circulating and tumor-infiltrating neutrophils is correlated with glioma grade (87–89). In GBM, clinical data suggests that an elevated level of neutrophils is negatively correlated with patients’ prognosis (90, 91). It was found that the stimulation of neutrophils was correlated with elevated levels of IL-12 in GBM patients, which was considered an early sign of tumor progression (87). Moreover, GBM patients with high levels of stimulated neutrophils were found to have a worse prognosis than those with low levels (91). Accordingly, the stimulation of neutrophils also has a negative prognostic value for GBM patients. In addition, the neutrophil-to-lymphocyte ratio (NLR) in blood is positively related to glioma grade, and elevated baseline NLRs predict poor prognosis in GBM patients (88, 92–94).

Neutrophils can be recruited into the TME by various cytokines, such as IL8, MIF, and CXCL8, which in turn leads to aggressive tumor growth and therapeutic resistance in GBM (95–97). Once infiltrated into the TME, neutrophils can secrete elastase, which further facilitates neutrophil infiltration and promotes GBM cell invasion. For example, neutrophil infiltration can increase the expression of S100A4, which mediates the tumor progression with mesenchymal characteristics, favoring glioma invasion and resistance to anti-VEGF therapies (98). In addition to this, neutrophils can form neutrophil extracellular traps (NETs) to protect glioma cells and facilitate tumor progression (99). A recent study showed that radiation-induced senescence in GBM cells promoted the recruitment of Ly6G+ (TANs) through NFκB signaling (100, 101). However, these infiltrated Ly6G^+^ cells can in return support the conversion of GBM cells to glioma stem cells (GSCs) through dedifferentiation and nitrosative stress signaling (102). This mechanism was confirmed by the fact that Ly6G-neutralizing antibodies and NFκB inhibitors reduced GSCs and prolonged the survival of GBM-bearing mice (101). In addition, neutrophils can mediate tumor cell ferroptosis by inducing iron-dependent accumulation of lipid peroxides through transferring myeloperoxidase-containing granules into tumor cells. This ferroptosis induced by neutrophils was identified as a pro-tumorigenic role in GBM because of its promotion of tumor necrosis (103). Recently, results from CIBERSORT analyzing immune cell fractions among the molecular subtypes of GBM showed that mesenchymal GBM had a significantly elevated level of TANs compared with other subtypes, implying a potential role in the extreme immunosuppression of the TME and plausibly mediating immunotherapy resistance (104).

**Figure 2:**
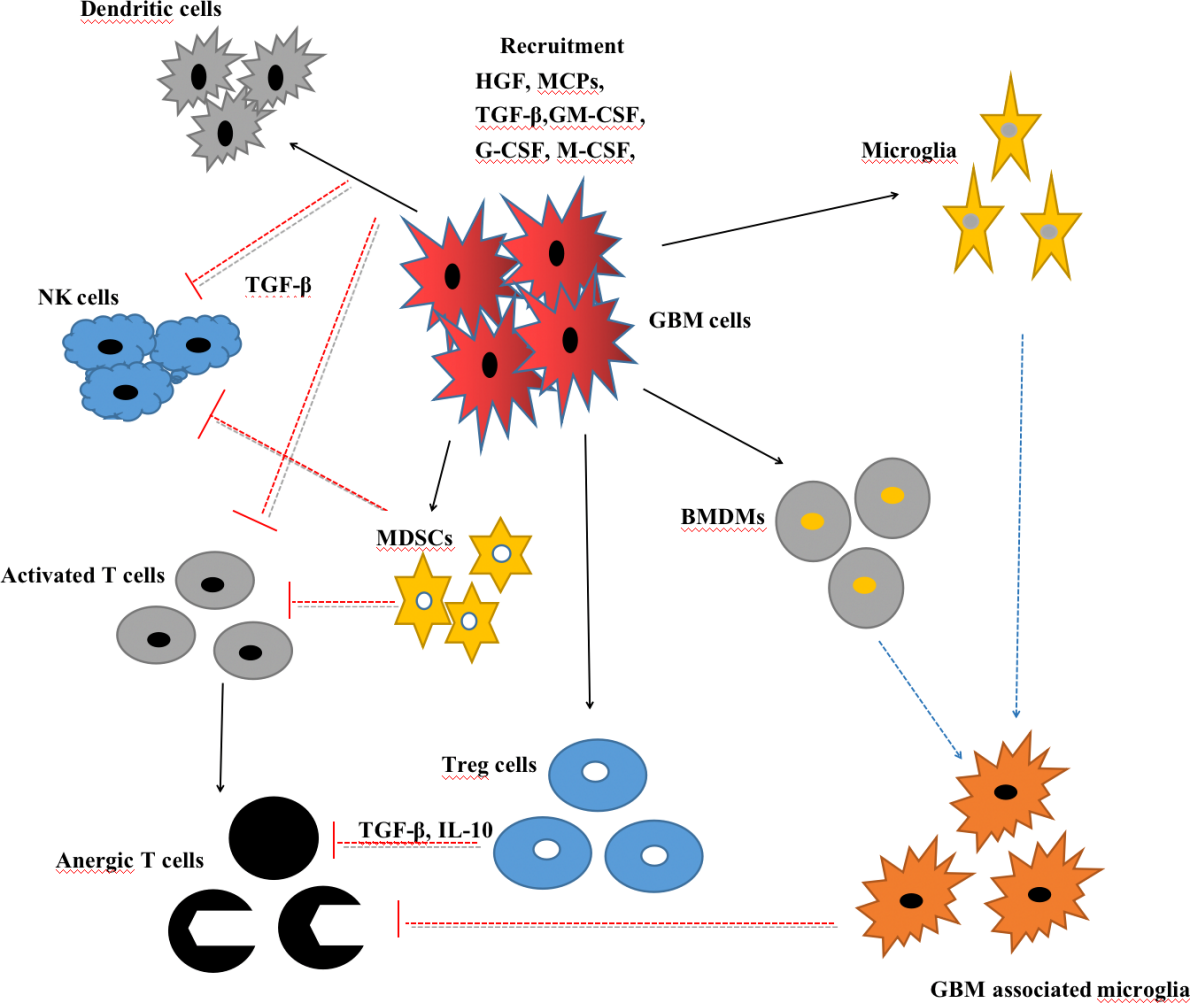
Interactions between glioblastoma (GBM) cells and myeloid cells, including microglia, GBM-associated microglia, bone marrow-derived macrophages (BMDMs), myeloid-derived suppressor cells (MDSCs), T cells, natural killer (NK) cells, and regulatory T cells (Tregs).

Taken together, circulating neutrophils and TANs are generally related to invasive tumor proliferation, tumor immunosuppression, and the poor prognosis of GBM. Further investigations are needed to explore the detailed mechanisms and establish a therapeutic strategy based on the neutrophils (**Figure 2**).

## 3 Current immunotherapy against GBM

The goal of immunotherapy is to harness the hosts’ innate and adaptive immune system by enhancing or suppressing immune responses to promote cancer eradication and overcome cancer’s immune resistance. Recently, immunotherapy has obtained great achievements in melanoma, renal cell carcinoma, Hodgkin’s lymphoma, and non-small cell lung cancer (NSCLC), in which conventional therapies have gained limited success (105–108). With the 2018 Nobel Prize in Medicine awarded to Tasuku Honjo and James Allison for their discovery of the inhibition of negative immune regulation in cancer therapy, immunotherapy has now taken the leading position in cancer therapies (12). In GBM, several immunotherapies including vaccine therapies, ICIs therapies, oncolytic virus therapy, and adoptive T-cell therapy have been investigated alone or combined with standard treatment in preclinical and clinical research.

### 3.1 Vaccine therapy

The vaccine therapy, which aims to enhance the adoptive immune response in the brain against GBM cells, generally involves two forms of therapies: peptide vaccines and DC vaccines. Peptide vaccines are generally designed to encompass tumor-specific antigens (TSAs) or tumor-associated antigens (TAAs) to induce a potent immunity. Currently, peptide vaccines that target a single GBM antigen include EGFRvIII, IDH1^R132H^, Wilms’ tumor 1 (WT1), and survivin. Results from phase II clinical trials have demonstrated that rindopepimut, an EGFRvIII-based vaccine, has a beneficial role in improving progression-free survival (PFS) and median overall survival (OS) in both primary and recurrent GBM patients (109, 110). However, no such therapeutic benefit of rindopepimut was observed in a randomized phase III clinical trial among newly diagnosed GBM (ndGBM) patients (111). Similar to EGFRvIII, the WT1-based vaccine has also shown benefits in improving GBM patients’ survival in a non-randomized trial (112). Recently, a novel vaccine named IMA950 that contains nine synthetic tumor-associated HLA-A2-restricted peptides (TUMAP), and thus can trigger the TUMAP-specific cytotoxic T cells, has shown therapeutic effect in improving the PFS and OS of ndGBM patients in phase I/II clinical trials (113, 114). Further evidence from phase III clinical trials is still lacking. GBM is notorious for its high heterogeneity and low mutational burden (115); thus, vaccines that target a single tumor antigen may theoretically lead to antigen escape. This was confirmed by the phenomenon in the EGFRvIII vaccine research, in which most patients experiencing recurrence had lost EGFRvIII expression (116). Therefore, alternative vaccine strategies that target multiple tumor neoantigens are needed. Heat shock protein (HSP) peptide complex 96 (HSPPC-96), a primary resident chaperone of the endoplasmic reticulum that can be internalized into antigen presenting cells (APCs) for efficient class I and II MHC-mediated presentation of tumor peptides, is a solution to this problem. In phase I and II clinical trials, HSSPC-96 vaccination showed a therapeutic effect in improving the OS in high-grade glioma and GBM patients (117). More evidence from phase III clinical trials is needed.

Dendritic cell (DC) vaccines are typically produced through the *ex vivo* generation of DCs harvested from patients and stimulated with either tumor antigens or mRNA-expressing MHC molecules, before being administered back to patients (118, 119). Similar to the peptide vaccine, the DC vaccines can also be loaded with a single tumor antigen or multiple tumor antigens. A WT1- and cytomegalovirus phosphoprotein 65 RNA (CMV pp65)-based DC vaccine is generated from a single tumor antigen, both of which have demonstrated efficiency in improving the PFS and OS of GBM patients in phase I clinical trials (120–123). On the other hand, DC vaccines loaded with multiple tumor antigens are, in theory, thought to induce more robust immune responses. ICT-107 is a DC vaccine loaded with class I peptides from a TAA highly expressed on gliomas and glioma stem cells (GSCs). It presented a survival benefit in a phase I clinical trial of 15 GBM patients, among whom six patients showed no evidence of tumor recurrence at a follow-up of over 40 months (124). Another α-type 1 polarized DC vaccine loaded with EphA2, IL13Ra2, YKL-40, and gp100 also showed survival benefits for recurrent glioma patients, with one patient achieving a sustained complete response (125). In addition, a novel DC vaccine named DCVax-L, which is loaded with tumor lysates, has shown survival benefits for GBM patients in a phase I/II clinical trial. Phase III clinical trial of DCVax-L in GBM patients is ongoing, and preliminary results indicate that it may improve patients’ OS (126).

### 3.2 Adoptive T-cell therapy

The primary forms of adoptive T-cell therapy can be generally classified as T-cell receptor (TCR) treatment, tumor-infiltrating lymphocytes (TILs), and chimeric antigen receptor T (CAR-T) cells. TCR treatment is the first successful application of an adoptive T-cell therapy, which utilizes autologous T cells transduced with human TCR to target a melanoma antigen recognized by T cells 1 (MART-1) in order to treat metastatic melanoma patients (127). However, no achievement has been obtained in clinical trials based on TCR-T cell treatment. In addition, the administration of autologous TILs has induced regressions in metastatic melanoma patients (128); however, such powerful cytotoxicity was not seen in GBM patients (129). In fact, the application of TILs requires highly accessible and immunogenic tumor cells. Melanoma can meet sufficient isolation and expansion of TILs from their respective tumor samples, but cannot (128) when it comes to GBM characterized by a high heterogeneity and a low mutational burden. The limited progress made in TCR and TILs against GBM has necessitated the development of CAR-T cell therapy.

Genetically engineered T cells express chimeric antigen receptors (CARs), which consist of an intracellular T-cell signaling domain with one or more single-chain variable fragments (scFvs) and an extracellular antigen recognition domain to target specific neoplastic cells (130, 131). The advantages of CAR-T cells lie in not only the recognition of specific antigens and stimulation without MHC limitation, but also their capability of generating an anti-tumor immune microenvironment featuring decreased levels of anti-inflammatory factors and increased levels of pro-inflammatory factors (132, 133). Recently, CD19-specific CAR-T cell therapy has shown excellent therapeutic safety and efficacy in hematological malignancies and solid cancers, and is therefore preferred in clinical treatment (134–139). Owing to these advances in other kinds of malignancies, CAR-T cell therapy is of extreme interest in GBM treatment and can be applied intravenously, intracranially, or intralesionally (140). So far, results from clinical trials of GBM patients are available for CAR-T cells targeting three antigens: EGFRvIII, IL-13 receptor alpha 2(IL-13Rα2), and human epidermal growth factor receptor 2 (HER2) (141–144). EGFRvIII is supposed to enhance GBM proliferation, angiogenesis, and invasiveness (145), and IL-13Rα2 is associated with GBM invasiveness and poor prognosis; both are GBM-specific antigens (146, 147). HER2 is a GBM-associated antigen that has been identified as an independent unfavorable

prognostic indicator for GBM patients (148). CAR-T cells that target these three antigens showed feasibility, safety, and potential efficiency in early clinical trials against GBM (141–144). However, CAR-T cell therapy is still faced with several intractable obstacles to the treatment of GBM. First, the non-uniform antigen expression resulting from the high heterogeneity of GBM will lead to antigen escape and tumor recurrence. For example, a recurrent GBM (rGBM) patient obtained tumor intracranial and spinal regression after intracranial administration of IL-13Rα2-targeted CAR-T cells, but IL-13Rα2-negative tumors resulted in a subsequent relapse (147). Second, CAR-T cell proliferation and persistence *in vitro* and CAR-T exhaustion in the TME is also an intractable problem that requires maximal sustenance of CAR-T cells. Last, the CAR-T cells’ administration may trigger some resistant mechanisms, including up-regulation of immunosuppressive factors (e.g., IDO1, PD-L1) and recruitment of immunosuppressive cells (e.g., Tregs, MDSCs) (140, 149). These obstacles require CAR-T cell therapy to target multiple antigens or to be combined with other therapies. Hegde et al. successfully created bivalent CAR-T cells (targeting HER2 and IL-13Rα2) and trivalent CAR-T cells (targeting HER2, IL-13Rα2, and EphA2), both of which showed more efficacy in preclinical studies than monovalent CAR-T cells (150–152). Notably, trivalent CAR-T cells can capture nearly 100% of tumor cells and exhibit superior anti-tumor efficacy in a murine model (151). That is, to deal with the immunosuppressive TME, researchers have exploited the advantages of an oncolytic virus that has been demonstrated to enhance anti-tumor immunity and regulate the TME to improve CAR-T cells for solid tumor therapy (152, 153). Recently, researchers have successfully promoted *de novo* CD19-CAR-T cells to target solid tumor cells by tumor-selective delivery of oncolytic viruses encoded a truncated CD19 protein (154).

### 3.3 Immune checkpoint inhibitors therapy

Immune checkpoint inhibitors (ICIs) are antibodies that block the endogenous negative regulatory pathways mediated by immune checkpoints to reduce their inhibiting effects on T-cell stimulation, proliferation, and function. Currently, ICIs that target cytotoxic T lymphocyte-associated antigen 4 (CTLA4) or programmed cell death protein 1 and its ligand (PD-1 and PD-L1) have been approved by the US Food and Drug Administration (FDA) for clinical application owing to their notable successes against melanoma and other solid malignancies (155–159). For the treatment of GBM, results from preclinical studies are encouraging; however, clinical success is still lacking (160–162). Recently, three phase III clinical trials (CheckMate 143, 498, and 548) investigated nivolumab, a PD-1 monoclonal antibody, alone or combined with another therapy for the treatment of rGBM or ndGBM patients. All these trials have declared a failure to meet the primary end point of OS, although some results await publication (163, 164).

Multifarious factors attenuate the efficiency of the PD-1 inhibitor in treating GBM patients, including extensive tumor heterogeneity, low tumor mutation burden (TMB), T-cell dysfunction and exhaustion, and DNA mismatch repair (dMMR) system status (165–167). The extensive tumor heterogeneity will lead to a heterogeneous expression of PD-1 in GBM, which is negatively correlated with patient prognosis (168). Recent studies show that tumors with high TMB are more responsive to ICIs (169, 170). In GBM, high TMB usually arises late after the extended application of chemotherapy. This suggests that patients with recurrent malignancies who have accepted chemotherapy may be more responsive to ICIs, and TMB should be regarded as a biomarker to select patients who are more likely to benefit from ICIs. Accordingly, the FDA has identified a high level of TMB [≥ 10 mutations per megabase (mut/mb)] as one of the inclusion criteria for choosing patients with malignancies for ICIs therapy, including those with gliomas (171). Clinical data show that only a subgroup of GBM patients has benefited from ICIs therapy and obtained prolonged survival (172). For example, in a phase II clinical trial, 35 rGBM patients accepted pembrolizumab (PD-1 monoclonal antibody) as neoadjuvant or adjuvant-only therapy. Patients accepting neoadjuvant pembrolizumab showed a statistically significant increase in OS and PFS compared with those in the adjuvant group. It was also demonstrated that neoadjuvant ICIs therapy was related to a tumor-specific up-regulation of the IFN-g-responsive gene signature and a declined cell cycle-related gene signature in the tumor (173). Moreover, another factor that limits the efficacy of ICIs therapy is T-cell dysfunction and exhaustion, which has long been identified as a hallmark of GBM (174–176). Apart from the PD-1/PD-L1 pathway, GBM is characterized by the up-regulation of multiple alternative immune checkpoints such as T-cell immunoglobulin and mucin domain 3 (TIM-3), B7 homolog 3 (B7-H3), indoleamine 2,3-dioxygenase-1 (IDO1), and lymphocyte activation gene 3 (LAG3), which usually mediate T transition into a dysfunctional, exhausted status (177–179). From this perspective, multitargeted inhibitors or combined ICIs therapies may theoretically be more likely to avoid T-cell dysfunction and exhaustion. Recently, a preclinical study has highlighted a possible combination of PD1, LAG3, and TIM3 blockade in a glioma murine model (180). Furthermore, antibodies targeting TIM-3 and LAG3 either alone or combined with anti-PD1 blockade are being tested among GBM patients clinically (NCT02658981, NCT02817633). In addition, the DNA mismatch repair (dMMR) system status, which is responsible for correcting DNA mismatches during replication to maintain DNA stability, is also associated with the efficacy of ICIs therapy (181). It has been demonstrated that GBM with dMMR deficiency is correlated with high TMB and overexpressed neoantigens, which are usually identified as “immunoresponsive” (182). Clinically, high-grade glioma patients without dMMR expression showed statistically longer OS and PFS after pembrolizumab administration than those with weak dMMR expression, indicating that patients without dMMR expression may benefit from pembrolizumab monotherapy (183) (**Table 1**).

**Table 1:** Ongoing clinical trials involving immune checkpoint inhibitors (ICIs), vaccines, and adaptive T cells. .

Status/Phase	Study Title		Details			NCT Number	
ICIs therapy							
I/II	Radiation Therapy with TMZ and Pembrolizumab in Treating Patients with Newly Diagnosed GBM		Radiation therapy, TMZ, pembrolizumab			NCT02530502
1/II	Pharmacodynamic Study of Pembrolizumab in Patients with Recurrent GBM		Pembrolizumab			NCT02337686
II	Pembrolizumab +/- Bevacizumab for Recurrent GBM		Pembrolizumab, bevacizumab			NCT02337491
1/I	Parameters For The PD-1 Checkpoint Inhibitor, Pembrolizumab (MK-3475), in Patients with Surgically Accessible Recurrent/Progressive Glioblastoma		Pembrolizumab			NCT02852655
1/I	Avelumab in Patients with Newly Diagnosed Glioblastoma Multiforme		Avelumab			NCT03047473
1/II	Avelumab With Hypofractionated Radiation Therapy in Adults with Isocitrate Dehydrogenase (IDH) Mutant Glioblastoma		Avelumab, radiotherapy			NCT02968940
1/II	Tremelimumab and Durvalumab in Combination or Alone in Treating Patients with Recurrent Malignant Glioma		Durvalumab, tremelimumab			NCT02794883
1/II	Combination Adenovirus + Pembrolizumab to Trigger Immune Virus Effects		Pembrolizumab, oncolytic virus			NCT02798406
1/II	Tremelimumab and Durvalumab in Combination or Alone in Treating Patients with Recurrent Malignant Glioma		Durvalumab + tremelimumab			NCT02794883
2/II	Phase 2 Study of Durvalumab (MEDI4736) in Patients with Glioblastoma		Durvalumab, TMZ, bevacizumab, radiotherapy			NCT02336165
4/I	Pembrolizumab and Vorinostat Combined with Temozolomide for Newly Diagnosed Glioblastoma		Pembrolizumab, vorinostat, TMZ, radiotherapy			NCT03426891
4/III	An Investigational Immuno-Therapy Study of Temozolomide Plus Radiation Therapy with Nivolumab or Placebo, for Newly Diagnosed Patients with Glioblastoma		Nivolumab, TMZ, radiotherapy			NCT02667587
4/III	An Investigational Immuno-Therapy Study of Nivolumab Compared to Temozolomide, Each Given with Radiation Therapy, for Newly diagnosed Patients with Glioblastoma		Nivolumab, TMZ, radiotherapy			NCT02617589
4/I	Biomarker-Driven Therapy Using Immune Activators with Nivolumab in Patients with First Recurrence of Glioblastoma		Nivolumab + anti-GITR monoclonal antibody, IDO1 inhibitor, ipilimumab			NCT03707457
4/I	Nivolumab, BMS-986205, and Radiation Therapy with or without Temozolomide in Treating Patients with Newly Diagnosed Glioblastoma		Nivolumab, IDO1 inhibitor			NCT04047706
4/II	Atezolizumab in Combination with Temozolomide and Radiation Therapy in Treating Patients with Newly Diagnosed Glioblastoma		Atezolizumab, radiotherapy, TMZ			NCT03174197
4/I	Avelumab With Laser Interstitial Therapy for Recurrent Glioblastoma		Avelumab, MRI-guided LITT therapy			NCT03341806
4/I	GMCI, Nivolumab, and Radiation Therapy in Treating Patients with Newly Diagnosed High-Grade Gliomas		Nivolumab, radiotherapy, oncolytic virus, TMZ			NCT03576612
Vaccine therapy							
1/I	Vaccine Therapy in Treating Patients with Newly Diagnosed Glioblastoma Multiforme		Autologous DCs, tetanus toxoid, therapeutic autologous lymphocytes			NCT00639639
1/II	Dendritic Cell Vaccine for Patients with Brain Tumors		DCs vaccination, adjuvant poly-ICLC			NCT01204684
1/I	Phase I Study of a Dendritic Cell Vaccine for Patients with Either Newly Diagnosed or Recurrent Glioblastoma		DCs vaccine, radiotherapy, TMZ, bevacizumab			NCT02010606
3/II/III	Proteome-Based Personalized Immunotherapy of Glioblastoma		DCs vaccine, allogeneic hematopoietic stem cells, cytotoxic lymphocytes			NCT01759810
4/I/II	Adjuvant Dendritic Cell-immunotherapy Plus Temozolomide in Glioblastoma Patients		DCs vaccine, TMZ			NCT02649582
4/II	Immunotherapy Targeted Against Cytomegalovirus in Patients with Newly Diagnosed WHO Grade IV Unmethylated Glioma		Human CMV pp65-LAMP mRNA-pulsed autologous DCs containing GM CSF, TMZ, tetanus–diphtheria toxoid (Td)			NCT03927222
4/II	Study of DC Vaccination Against Glioblastoma		DCs vaccine, radiotherapy, TMZ			NCT01567202
4/I	Pembrolizumab and a Vaccine (ATL-DC) for the Treatment of Surgically Accessible Recurrent Glioblastoma		DCs vaccine, pembrolizumab, poly-ICLC			NCT04201873
I	Nivolumab With DC Vaccines for Recurrent Brain Tumors		DCs vaccine, nivolumab			NCT02529072
4/II	Radiation Therapy Plus Temozolomide and Pembrolizumab with and without HSPPC-96 in Newly Diagnosed Glioblastoma (GBM)		HSPPC-96, pembrolizumab, TMZ			NCT03018288
4/II	Efficiency of Vaccination with Lysate-loaded Dendritic Cells in Patients with Newly Diagnosed Glioblastoma		DCs vaccine, radiotherapy, TMZ			NCT03395587
4/I	Safety and Immunogenicity of Personalized Genomic Vaccine and Tumor Treating Fields (TTFields) to Treat Glioblastoma		Peptides vaccine, poly-ICLC, tumor treating fields			NCT03223103
4/I/II	Study to Evaluate Safety, Tolerability, and Optimal Dose of Candidate GBM Vaccine VBI-1901 in Recurrent GBM Subjects		VBI-1901 (a polyvalent therapeutic vaccine against cytomegalovirus antigen gB and pp65) + GM-CSF			NCT03382977
Adaptive T-cell therapy							
4/I	Genetically Modified T-cells in Treating Patients with Recurrent or Refractory Malignant Glioma		IL13Rα2-specific, 41BB-costimulatory CAR/truncated CD19-expressing autologous T lymphocytes			NCT02208362
4/I	IL13Ralpha2-Targeted Chimeric Antigen Receptor (CAR) T Cells with or without Nivolumab and Ipilimumab in Treating Patients with Recurrent or Refractory Glioblastoma		IL13Rα2-specific hinge-optimized 4–1BB-costimulatory CAR/truncated CD19-expressing autologous TN/MEM cells, ipilimumab, nivolumab			NCT04003649
4/I	Combination of Immunization and Radiotherapy for Malignant Gliomas		GM-CSF + poly-I:C or CAR-T or TCR-T + radiation			NCT03392545
I/II	CAR-T Cell Receptor Immunotherapy Targeting EGFRvIII for Patients with Malignant Gliomas Expressing EGFRvIII Recruiting		EGFRvIII targeted CAR-T cell			NCT01454596
I/II	CAR-T Cell Immunotherapy in MUC1 Positive Solid Tumor		Anti-MUC1 CAR-T cells			NCT02617134
I/II	CAR-pNK Cell Immunotherapy in MUC1 Positive Relapsed or Refractory Solid Tumor		Anti-MUC1 CAR-pNK cells			NCT02839954
I/II	A Clinical Research of CAR T Cells Targeting HER2 Positive Cancer		HER2-targeted CAR-T cell			NCT02713984
I	Autologous T Cells Redirected to EGFRVIII-With a CAR in Patients With EGFRVIII+ Glioblastoma		EGFRvIII-targeted CAR-T cell			NCT02209376
I	EGFRvIII CAR T Cells for Newly Diagnosed GBM		EGFRvIII-targeted CAR-T cell			NCT02664363
I	Pilot Study of Autologous Anti-EGFRvIII CAR T Cells in Recurrent Glioblastoma Multiforme		EGFRvIII-targeted CAR-T cell			NCT02844062
I	CMV-specific Cytotoxic T Lymphocytes Expressing CAR Targeting HER2 in Patients with GBM		HER2-targeted CAR-T cell			NCT01109095
I	T Cells Expressing HER2-specific CAR for Patients With Glioblastoma		HER2-targeted CAR-T cell			NCT02442297
I	Pilot Study of Autologous Chimeric Switch Receptor Modified T Cells in Recurrent GBM		PD-L1-target CAR-T cell			NCT02937844

CAR, chimeric antigen receptor; CAR-T chimeric antigen receptor T; CMV pp65, cytomegalovirus phosphoprotein 65; DC, dendritic cell; EGFR, epidermal growth factor receptor; HER2, human epidermal growth factor receptor 2; HSPPC-96, heat shock protein peptide complexes 96; IDO1, indoleamine 2,3-dioxygenase; NCT, National Clinical Trial; PD-L1, program death 1 ligand. TMZ, temozolomide; pp65-LAMP, pp65-lysosomal-associated membrane protein poly; ICLC, Polyinosinic-polycytidylic acid-poly-l-lysine carboxymethylcellulose; MRI-guided LITT therapy, magnetic resonance-guided laser interstitial thermal therapy; GM CSF, granulocyte-macrophage colony-stimulating factor; TN/MEM cells, naïve and memory T cells; TCR-T, T cell receptor-T cell; Poly I:C:Polyinosinic polycytidylic acid; MUC1, mucin 1.

### 3.4 Oncolytic virotherapy

Oncolytic virus (OV) therapy is a novel and promising therapeutic remedy in

the treatment of various solid tumors, including GBM. It can be divided into two broad categories: replication-competent viruses, which function as viral vectors to introduce specific genes, such as tumor suppressor genes, suicide genes, or immunostimulatory genes, into tumor cells to trigger an anti-tumor response; and selectively replication-competent viruses, which infect tumor cells and replicate until the cell lyses, and then infect and lyse neighboring cells (1–3, 184–186). Thus, the anti-tumor effect introduced by OVs occurs through two mechanisms: the direct killing of tumor cells and the subsequent stimulation of innate and adaptive immune responses. After the direct tumor lysis and apoptosis introduced by OVs, TAAs, pathogen-associated molecular patterns (PAMPs), and damage-associated molecular patterns (DAMPs) are released from the disrupted tumor cells. Both PAMPs and DAMPs can not only trigger innate immunity by stimulating pattern recognition receptors but also improve antigen cross-presentation and adaptive immune responses (4, 5, 187, 188). Therefore, OVs are also thought to be capable of turning the TME from immunologically “cold” to “hot”.

Oncolytic virus (OV) therapy has been investigated for the treatment of malignancies for decades. In 2015, the FDA approved the application of talimogene laherparepvec (T-VEC), a genetically modified Herpes Simplex Virus (HSV), to treat advanced melanoma as the first OV therapy in the USA (6, 189). GBM virotherapy clinical trials started in 1991 (7, 190). Since then, multifarious genetically engineered OV candidates have been investigated to treat GBM both in preclinical and clinical research. These include parvovirus, oncolytic herpes simplex virus (oHSV1716, oHSVG207), conditionally replicating adenovirus (ONYX-015, DNX-2401, DNX-2440), measles paramyxovirus (MV-CEA, MV expressing IL-13), oncolytic polio/rhinovirus recombinant (PVSRIPO), and retroviral replicating vector (Toca 511). Among these, DNX-2401, PVSRIPO, and Toca 511 are the most promising, having shown complete durable responses when intratumorally administered in GBM patients (8–10, 191–193). However, these trials failed to meet primary end points. Recently, the novel strategy of inserting immunomodulatory genes into the viral genome to conduct OVs to express immunomodulatory transgenes has been evaluated in several preclinical trials to treat gliomas. These include OVs with an expression of IL-12, IL-15, ICIs, tumor necrosis factor (TNF)-related apoptosis-inducing ligand (TRAIL), immune stimulators (GM-CSF, OX40 ligand), tumor suppressors (PTEN, P53), E-cadherin, and FMS-like tyrosine kinase 3 ligand (Flt3L) (11–20, 152, 194–202). Some have demonstrated safety and efficacy in preclinical trials, but others still require further investigation.

Although OV therapy has drawn concentrations, powerful clinical effectiveness in treating GBM is still lacking. The first obstacle is the limited efficiency of OV delivery. Several factors impact its systemic delivery, including complement factor neutralization, off-target dispersion risk, virus reduction during hepatic metabolism, and limitation from the BBB. To address these problems, mesenchymal stem cells (MSCs) with tumor-tropic and immune-evasive abilities are introduced as carriers for the virus delivery. Not only can MSCs shield OVs from host immunity, but they also alleviate the systemic sequestration of viruses and off-target toxicities. A recent study has successfully conducted MSCs to migrate and deliver therapeutic viruses to distant glioma cells (21, 203). That is, most trials concerning GBM adopted intracranial or intratumoral administration of OVs to overcome the BBB. However, it is also intractable for the second obstacle introduced by the immunosuppressive TME. Recent studies have demonstrated that GSCs and glioma-associated mesenchymal stem cells in the TME are likely to generate resistance to OV therapy (22, 23, 204, 205). Moreover, it has been demonstrated that TAMs can restrict the replication and spread of oncolytic viruses (24, 206). Furthermore, MDSCs can induce B-cell-mediated immunosuppression, which may limit the effective response to OVs with the expression of immunomodulatory transgenes (25, 81). Accordingly, a better-designed delivery and a better understanding of the TME should be pursued to optimize OV therapy against GBM.

## 4 Conclusion

Myeloid cells, including BMDMs, microglia, MDSCs, and neutrophils, play a crucial role in maintaining the immunosuppressive TME in GBM. Numerous factors impact the recruitment, stimulation, and function of myeloid cells in the TME, which constitutes a significant challenge for therapy efficacy. Currently, immunotherapies predominantly focus on the investigation of vaccines, CAR-T cells, ICIs, and OVs therapies. However, successful advances in a clinical environment are still lacking. Moreover, single immunotherapy is always insufficient in GBM and leads to the formation of a “cold tumor”, because multifarious factors challenge immunotherapy, including the immunosuppressive TME, the high tumor heterogeneity, the low TMB, the dMMR status, persistence and delivery of the vaccines, and CAR-T cells. Thus, a better understanding of the functions of myeloid cells in the TME and their interactions with GBM cells should be pursued in the future to optimize therapeutic strategy. Recently, combined therapy of OVs with ICIs in GBM demonstrated an improved efficiency over OV therapy alone (207). Similar results have also been obtained from the combined therapy of OVs with CAR-T cell therapy compared with CAR-T cells alone (154). Thus, this combination should be further investigated in order to be applied in clinical environments. Accordingly, immunotherapy for GBM requires integrated efforts to reduce and deactivate immunosuppressive myeloid cells, improve the TME suppression, promote CAR-T cell function by equipping multi-antigens targets, and reinforce T-cell effector function with the blockade of immune checkpoints. These contributions will promote the development of an optimal personalized therapeutic strategy for GBM patients.

## Author contributions

BH and JZ wrote the initial draft. Figures were prepared by WZ. HZ prepared the final version. ZZ recommended a structure for the review and substantially advanced the draft. All authors contributed to the article and approved the submitted version.
